# Preparation of Stable Silver Nanoparticles Having Wide Red‐To‐Near‐Infrared Extinction

**DOI:** 10.1002/gch2.201700105

**Published:** 2018-02-21

**Authors:** Shiori Kawamura, Kazuki Matsubara, Sotaro Sakai, Kazuhisa Sasaki, Masataro Saito, Kenji Saito, Masayuki Yagi, Wataru Norimatsu, Ryo Sasai, Michiko Kusunoki, Miharu Eguchi, Shu Yin, Yusuke Asakura, Tatsuto Yui

**Affiliations:** ^1^ Department of Materials Science and Technology Faculty of Engineering Niigata University 8050 Ikarashi‐2 Niigata 950‐2181 Japan; ^2^ Department of Materials Chemistry Graduate School of Engineering Nagoya University Furo‐cho, Chikusa‐ku Nagoya‐shi Aichi‐ken 464‐8603 Japan; ^3^ Department of Physics and Materials Science Interdisciplinary Graduate School of Science and Engineering Shimane University 1060, Nishi‐kawatsu‐cho Matsue 690‐8504 Japan; ^4^ Institute of Materials and Systems for Sustainability Nagoya University Furo‐cho, Chikusa‐ku Nagoya‐shi Aichi‐ken 464‐8603 Japan; ^5^ Electronic Functional Materials Group Polymer Materials Unit National Institute for Materials Science (NIMS) 1‐1 Namiki Tsukuba Ibaraki 305‐0044 Japan; ^6^ Institute of Multidisciplinary Research for Advanced Materials Tohoku University 2‐1‐1, Katahira Aoba‐ku Sendai 980‐8577 Japan

**Keywords:** catalysts, layered materials, layered semiconductors, metal nanoparticles, near‐infrared

## Abstract

The synthesis of silver nanoparticles (AgNPs) within the interlayer space of transparent layered titania nanosheet (TNS) films is investigated. A considerable number of silver ions (≈70% against the cation exchange capacity of the TNS) are intercalated in the TNS films using methyl‐viologen‐containing TNSs as a precursor. The silver ion (Ag^+^)‐containing TNS films are treated with aqueous sodium tetrahydroborate (NaBH_4_), resulting in a gradual color change to bright blue. Various structural analyses clearly show that crystalline AgNPs are generated within the interlayer space of the TNSs. The NaBH_4_‐treated films show intense and characteristic near‐infrared (NIR) extinction spectra up to 1800 nm. The stability of the AgNPs within the TNS against oxygen and moisture is also investigated, and 96% and 82% of the AgNPs remain after standing in air for 1 month and 1 year, respectively. The NIR extinctions of the AgNP‐containing TNS films are further extended by employing different preparation procedures, for example, using sintered TNS films as starting materials and irradiating the Ag^+^‐containing TNSs with ultraviolet (UV) light. The obtained AgNP‐containing TNS films exhibit photochemical activities in the production of hydrogen from ammonia borane under visible‐light irradiation and the decomposition of nitrogen monoxide under UV‐light irradiation.

## 1 Introduction

Near‐infrared (NIR) responsive materials are expected to be applied in a wide range of research fields, especially the global challenging issues, e.g., health and medical applications including NIR imaging, solar energy conversion systems including hydrogen (H_2_) production from water, photocatalysts to remove carbon dioxide (CO_2_) that deteriorate the climate and to purify water or soil, as well as invisible security inks.^1^ Organic dyes and metal complexes have been developed as new types of NIR responsive, mainly NIR‐absorbing, materials.[[qv: 1h-j,2]] In order to endow a dye with NIR absorption, it is necessary to increase its highest occupied molecular orbital (HOMO) level and/or decrease its lowest unoccupied molecular orbital level of dyes. However, increasing the HOMO level enhances the oxidation of the material. Thus, many NIR‐absorbing organic dyes and metal complexes exhibit inferior durability. Consequently, the practical application of NIR‐responsive materials requires the development of air‐stable materials.

Metal nanoparticles (MNPs) exhibit characteristic light absorption and scattering owing to their localized surface plasmon resonance (LSPR), and MNPs have been applied as next‐generation photofunctional materials.^3^ Gold exhibits good stability against oxygen. Consequently, several NIR‐responsive materials comprising gold nanoparticles or other gold nanostructures have been reported.[[qv: 3f,g]] However, gold is relatively expensive. Therefore, the development of inexpensive MNPs with effective NIR responses is required.

Silver is relatively inexpensive, and is therefore attractive for this purpose. Consequently, silver nanoparticles (AgNPs) and their hybrids have been widely investigated.[[qv: 3h-k,4]] However, stable AgNP‐based NIR‐responsive materials that exhibit clear and intense NIR extinction (absorption and scattering) above 1200 nm have yet to be reported. For example, Tanaka and co‐workers reported the synthesis and NIR response of triangle‐shaped AgNPs. However, these AgNPs exhibited no extinction at wavelengths longer than 1100 nm.^5^ Yamashita and co‐workers reported the NIR response of AgNPs in mesoporous silica.[[qv: 3j]] However, they made no mention of NIR extinction above 1200 nm. Tatsuma and co‐workers reported the NIR response and photochromic properties of AgNPs and TiO_2_/AgNP hybrids.^6^ The TiO_2_/AgNP hybrids exhibited wide NIR extinction at <1700 nm. However, this system required complicated synthetic procedures and it exhibited low extinction intensities.[[qv: 6c,d]] Furthermore, all these reported materials have low or undisclosed stabilities in air.

Ogawa and co‐workers^7^ and our group^8^ have reported the in situ synthesis of various MNPs within the interlayer spaces of layered inorganic compounds. Our technique^8^ enables the formation of clear films having strong LSPR extinctions owing to the optical transparency of layered titania nanosheets (TNSs),^9^ and relatively large amounts of MNPs are accommodated in their interlayer space.

We repot here the synthesis of AgNPs within the interlayer spaces of TNSs and their unique NIR‐responsive properties. Sasaki and co‐workers have reported the photocatalytic reduction of Ag^+^ in the interlayer spaces of TNSs prepared using the layer‐by‐layer (LbL) technique.[[qv: 9d]] However, not only does the LbL technique involve complicated procedures and significant effort, the resultant films are very thin (≈20 layers of single TNSs). Thus, the optical properties and stabilities of the AgNPs prepared by this technique have not been investigated due to the thinness of the films.[[qv: 9d]] In contrast, the TNS and AgNP (TNS/AgNP) hybrids presented here exhibit wide and strong NIR responses and good stabilities, and are fabricated without complicated procedures. Moreover, the TNS/AgNP hybrids exhibit activity in photoassisted H_2_ production from amminetrihydridoboron (NH_3_BH_3_)[[qv: 3i,j]] and the photochemical decomposition of nitric oxide (NO).^10^ NH_3_BH_3_ is as an attractive material for next‐generation hydrogen‐storage systems,^11^ and the photochemical decomposition of NO is applicable to the removal of pollutants from automobile exhaust fumes.

## 2 Results and Discussion

### 2.1 Characterization of Ag^+^‐Intercalated TNS (TNS/Ag^+^) Films

**Figure 1:**
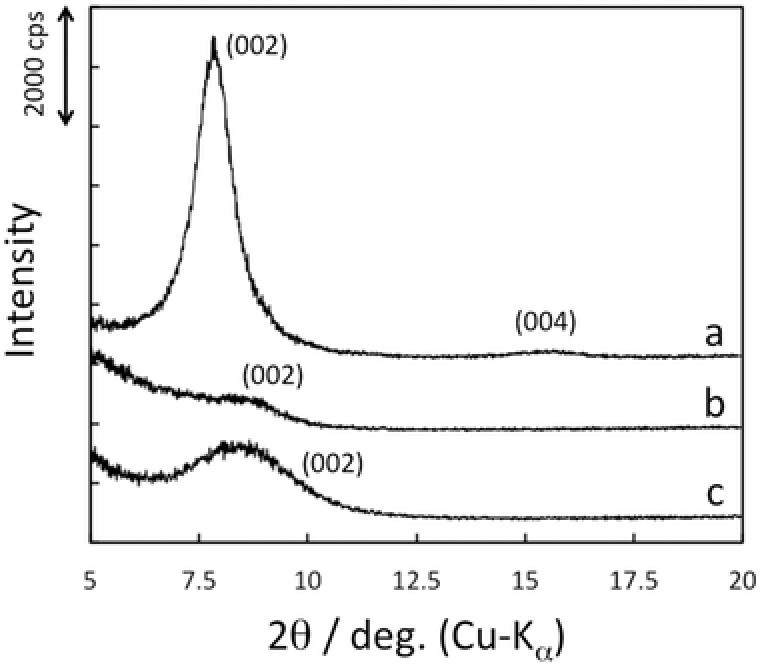
a) XRD profiles of TNS/MV^2+^, b) AgNO_3_‐treated TNS/MV^2+^ (TNS/Ag^+^), and c) NaBH_4_‐treated TNS/Ag^+^ (TNS/AgNP, where *N* = 1) films.

Intercalation of Ag^+^ into the TNS layers was performed by the treatment of methyl‐viologen (MV^2+^)‐intercalated films (TNS/MV^2+^) with aqueous silver nitrate (AgNO_3_)‐treated. The X‐ray diffraction (XRD) profiles of the TNS/MV^2+^ and AgNO_3_‐treated films are shown in **Figure** **1**a,b, respectively. Further details of the XRD analyses are summarized in Table S1 (Supporting Information). The initial TNS/MV^2+^ film exhibits clear *d*(002) and (004) signals at 7.8° and 15.3°, respectively, and this is in good agreement with the results of an earlier report.^8^ This result indicates that MV^2+^ molecules (molecular size: ≈1.3 × 0.4 nm) adopt almost parallel orientations in single layers. A very weak and broad XRD signal is observed at 8.7° (*d* = 1.01 nm) for the AgNO_3_‐treated films, indicating disorder of the layered structure. The thickness of one layer of the TNSs has been reported to be 0.75 nm.[[qv: 9b-e]] Thus, the clearance space (CLS) is 0.26 nm, which is close to the diameter of Ag^+^ (≈0.25 nm).^12^

The amounts of sodium (Na) and silver (Ag) adsorbed were determined by energy dispersive spectroscopy (EDS) analysis, and representative EDS spectra are shown in Figure S1a (Supporting Information). The atomic ratios of Ag and Na against titanium (Ti) and the adsorbed amounts of Ag and Na relative to the cation exchange capacity (CEC) of the TNSs are summarized in **Table** **1**. The CEC was estimated from the chemical structure of the starting material TNS (H_0.7_Ti_1.825_O_4_·H_2_O, where H^+^ (proton) is the exchangeable interlayer cation). A considerable amount of Ag atoms (≈72% vs CEC) and a very small amount of Na atoms (≈5% vs CEC) are detected in the AgNO_3_‐treated film, suggesting that the selective ion exchange of MV^2+^ with Ag^+^ proceeds successfully. Based on these results, we conclude that Ag^+^‐containing TNSs (TNS/Ag^+^) are formed using our experimental procedure.

**Table 1:** Atomic ratios of Ag and Na against Ti and adsorbed amounts of Ag and Na atoms against the cation exchange capacity of TNS (% vs CEC).

	Ag/Ti	Na/Ti	Amounts of Ag	Amounts of Na
TNS/Ag^+^	0.276 ± 0.012	0.020 ± 0.005	71.9 ± 3.1	5.3 ± 1.2
TNS/AgNP (*N* = 1)	0.280 ± 0.021	0.223 ± 0.017	73.1 ± 5.4	58.1 ± 4.4

### 2.2 Characterization of sodium tetrahydroborate (NaBH_4_)‐Treated TNS/Ag^+^

**Figure 2:**
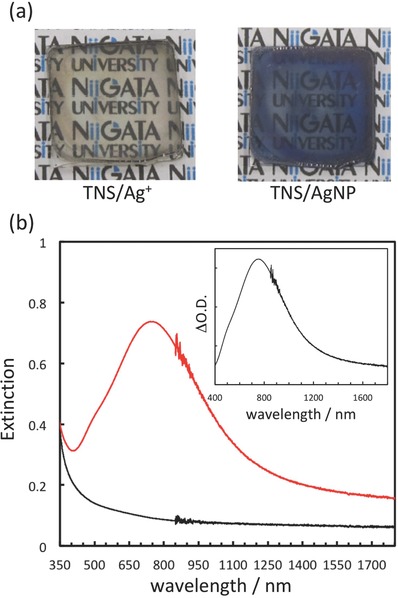
a) Photograph of a TNS/Ag^+^ film (left) and a NaBH_4_‐treated TNS/Ag^+^ (TNS/AgNP, *N* = 1) (right) film. b) Extinction spectra of TNS/Ag^+^ (black line) and NaBH_4_‐treated TNS/Ag^+^ (TNS/AgNP, *N* = 1) (red line) films. The inset shows the differential extinction spectra of the NaBH_4_‐treated TNS/Ag^+^ (TNS/AgNP) film. The spectrum was normalized by considering the spectrum of a TNS/Ag^+^ film as background and subtracting it from the spectra obtained after NaBH_4_ treatment.

Upon treatment of the TNS/Ag^+^ film with NaBH_4_ as reductant, the color of the film immediately changes from colorless to bright blue, as shown in **Figure** **2**a. A new extinction band[[qv: 4a]] at λ_max_ = 735 nm is observed following NaBH_4_ treatment (Figure 2b) and the NaBH_4_‐treated film exhibits clear NIR extinction up to 1800 nm. This color change indicates the reduction of Ag^+^ and the formation of AgNPs in the TNSs. We expect that such wide and nonsymmetrical extinction spectral shapes are due to the wide particle size and shape distributions of AgNPs in the TNSs, as described below.

**Figure 3:**
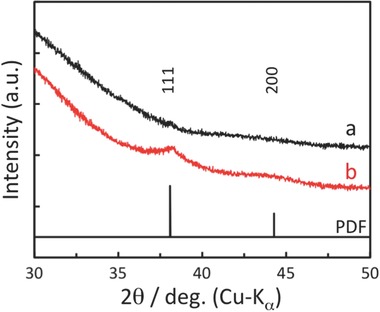
XRD profiles of a) TNS/Ag^+^ and b) NaBH_4_‐treated TNS/Ag^+^ (TNS/AgNP, *N* = 1) films at the high‐angle region, as well as the PDF index of crystalline Ag(0).

**Figure 4:**
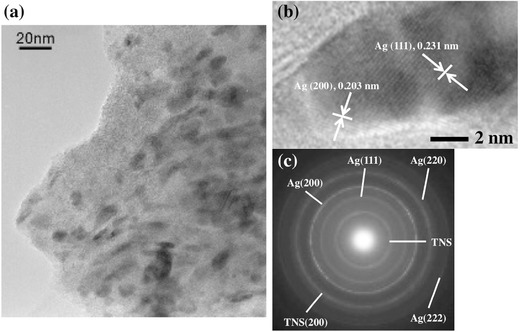
a,b) TEM images and c) electron diffraction pattern of the NaBH_4_‐treated TNS/Ag^+^ (TNS/AgNP, *N* = 1).

The XRD profiles of TNS/Ag^+^ and NaBH_4_‐treated TNS/Ag^+^ in the high‐angle region are shown in **Figure** **3**. The precursor TNS/Ag^+^ shows no diffraction signals in this region, while the NaBH_4_‐trated TNS/Ag^+^ shows characteristic (111) and (200) diffraction corresponding to Ag(0) crystals (PDF: 01‐071‐4613). Transmission electron microscopy (TEM) analysis clearly shows high‐contrast (i.e., heavier) oval‐shaped dots with wide size (5–20 nm) and shape distributions (**Figure** **4**a). High‐magnification TEM images show that the high‐contrast particles are sandwiched in TNS layers (Figure S2, Supporting Information). Furthermore, the high‐resolution TEM image of the dark dots clearly shows fringes with widths of 0.203 and 0.231 nm, corresponding to the spacings of (200) and (111) faces (Figure 4b), respectively. Moreover, clear diffraction patterns form crystalline Ag and TNS are observed in the electron diffraction (ED) analyses (Figure 4c).

The X‐ray photoelectron spectroscopy (XPS) spectra of NaBH_4_‐treated TNS/Ag^+^ (*N* = 1) and the corresponding peak energies are presented in Figure S3 and Table S2 (Supporting Information), respectively. The spectral shapes and peak energies from TNS (O 1s and Ti 2p) and Ag 3d are in good agreement with those in previous reports.[[qv: 9d]] The exact chemical structures of the Ag species were not identified owing to limitations in our apparatus resolution. Therefore, the chemical structures of these particles, i.e., Ag(0), Ag_2_O, and AgO, or their core–shell structures, have not yet been identified. However, once reduced, Ag(0) exhibits good stability against oxidation. Thus, the formation of oxidized Ag species is thought to be negligible. Based on the XRD results and the high‐resolution TEM, ED, and XPS analyses, we conclude that crystalline AgNPs are generated by NaBH_4_ treatment as the major component.

In order to confirm the formation of AgNPs within the interlayer space of the TNSs rather than just on the surface of the TNS film, the relationship between the amount of TNS on the substrate and the amount of AgNP was investigated. If the AgNPs are formed only on the surface of the films, the amounts of TNS and AgNPs should be independent.[[qv: 8a]] The absorption intensity at 380 nm for TNS/Ag^+^ corresponds to the amount of TNS on the substrate, and the extinction intensity at 800 nm for the NaBH_4_‐trated film corresponds to the amount of AgNPs, as shown in Figure S4 (Supporting Information). The extinction intensity of the AnNPs increases linearly with the increasing absorption intensity of TNS. This clearly indicates that AgNPs are formed in the interlayers of the TNSs rather than just on the surface of the films. EDS analysis shows that there is no significant difference in the amount of Ag atoms following NaBH_4_ treatment (Figure S1b, Supporting Information, and Table 1). Thus, Ag atoms are not desorbed from the TNS layers upon aqueous NaBH_4_ treatment. The amount of Na atoms clearly increases through NaBH_4_ treatment. This result strongly suggests that Na^+^ is intercalated accompanying the reduction of Ag^+^ in the interlayers of the TNSs due to charge compensation. The *d*(002) reflection signal in the XRD analysis is recovered through NaBH_4_ treatment (Figure 1c and Table S1, Supporting Information). However, almost no significant differences are observed for the *d*(002) diffraction angles of the starting TNS/Ag^+^ and NaBH_4_‐treated films. The TEM image clearly shows that Ag particles (5–20 nm) are distributed sparsely on the TNS surface (Figure 4a). However, the estimated particle size of Ag in the TNSs and the CLS of the NaBH_4_‐treated TNS/Ag^+^ (0.29 nm) are quite different. These results suggest that the ordering of the layer structure occurs due to intercalation of Na^+^ and that the XRD signals correspond to regions containing Na^+^. Conversely, the TNS layers in the vicinity of Ag particles are largely disordered and almost no diffractions are produced by these areas.

The proposed structures of the different TNS films are presented in Figure S5 (Supporting Information).^13^ Based on all these results, we conclude that the Ag^+^ in the TNS layers is effectively reduced and that nanoparticles of Ag(0), i.e., TNS/AgNP, are formed through NaBH_4_ treatment. As described in the Introduction section, there are few reports of AgNPs exhibiting clear NIR responses up to 1500 nm. Thus, the TNS/AgNP presented here may be considered as a new type of NIR‐responsive material.

### 2.3 Stability in Air

**Figure 5:**
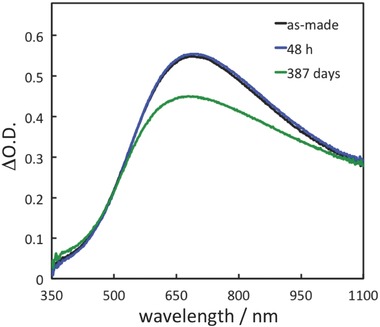
Differential extinction spectra of TNS/AgNP (*N* = 1) under ambient conditions (room temperature, air, and in the dark) for 0, 48 h, and 387 d.

To confirm the stability of TNS/AgNP in air, the TNS/AgNP films were stored in the dark and their extinction spectra were monitored. No significant differences in the extinction spectra are observed after 48 h (**Figure** **5**). Furthermore, there are no significant changes after one week, and only a slight decrease (4%) in extinction intensity is observed after 29 d. Moreover, 82% of the extinction intensity remains after 387 d. However, the extinction spectrum was slightly blue shifted after 1 year. This result suggests that AgNPs having different size and shape distributions show different oxidation resistance characteristics in the TNSs. Anyway, the origins of these changes are not clear, but we assume that the slow oxidation of the AgNP surfaces is a contributing factor.

There are several reports of blue‐colored AgNPs being easily oxidized by oxygen in the air without protective reagents. For example, Tanaka and co‐workers, reported the color change of triangular AgNPs in a colloidal solution as follows:^5^ When the colloidal solution was air‐bubbled, the color of the solution changed to green and then turned to colorless. When water was added to the photoirradiated solution, the solution immediately turned to light red and then colorless. However, the TNS/AgNP prepared in the present study exhibits stability against air and moisture for several hundred days.

### 2.4 Repeated NaBH_4_‐Treatment

**Figure 6:**
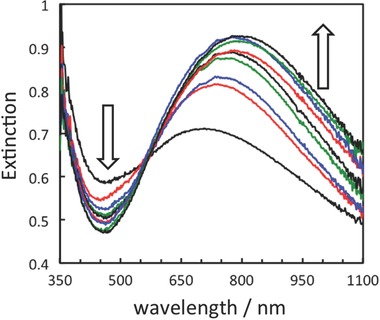
Changes in the extinction spectra of TNS/AgNP upon repeated NaBH_4_ treatment (*N* = 1–9).

In the above‐mentioned experiments, NaBH_4_ treatment of the TNS/Ag^+^ film was applied only once. However, it was suggested that the reduction of Ag^+^ did not proceed sufficiently. Therefore, NaBH_4_ treatment was performed multiple times, and the number of repetitions is denoted as *N*. The changes in the extinction spectra and extinction intensities at λ_max_ with different *N* values are shown in **Figure** **6** and Figure S6a (Supporting Information), respectively. With increasing *N*, an increase in extinction intensity at around 750 nm and a decrease in the intensity at 350–550 nm are observed. This indicates the growth of AgNPs within the TNS interlayers. Moreover, an increase in *N* induces a redshift in λ_max_ (Figure S6b, Supporting Information). These spectral changes become insignificant beyond *N* ≈ 5, suggesting that approximately five NaBH_4_ treatments are required to complete Ag^+^ reduction in the TNS layers under our experimental conditions.

**Figure 7:**
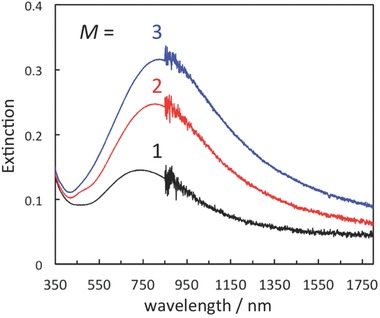
Changes in the extinction spectra of TNS/AgNP upon repeated AgNO_3_ then NaBH_4_ treatment (*M* = 1–3).

Based on the negative charge density (2.98–3.26 charge nm^−2^)^14^ of TNS, the negative charge distance of TNS is estimated to be ≈0.35 nm. This suggests that the Ag^+^ atoms are isolated in the interlayers of the TNSs, even at saturated adsorption (≈70% vs CEC). Moreover, abundant Ag^+^ atoms are transformed into one AgNP through reduction. Thus, it is expected that the density of the AgNPs will be very low in the TNSs (Figure 4a). However, further AgNPs can be introduced into the remaining interlayer space. To increase the amount of AgNPs, repeated sequential AgNO_3_ and NaBH_4_ treatments were investigated, and the number of repetitions is denotes as *M*. The extinction spectra of Ag‐containing TNS films prepared with different *M* values are shown in **Figure** **7**. Repeated AgNO_3_ then NaBH_4_ treatments induce an increase of the extinction band intensity at ≈750 nm. EDS analysis (Table S3, Supporting Information) clearly shows the linear relationship between the amount of Ag species and *M* (*M* × 70%). However, the amount of Ag species is not affected by NaBH_4_ treatment, indicating that the Ag species are not desorbed upon NaBH_4_ treatment. In contrast, the amount of Na repeatedly increases and decreases through repeated NaBH_4_ then AgNO_3_ treatment. However, the amount of Na is independent of *M*. Repeated disappearance and appearance of the *d*(002) reflection through AgNO_3_ and NaBH_4_ treatment is observed upon XRD analysis, as shown in Figure S7 (Supporting Information). These results strongly suggest that reintercalation of Na^+^ due to charge compensation accompanies the reduction of Ag^+^. To further increase the amount of AgNP and thus NIR absorption, we are currently investigating TNS/AgNPs prepared with *M* increased further, and the results will be reported in due course.

### 2.5 Extension of NIR Response

**Figure 8:**
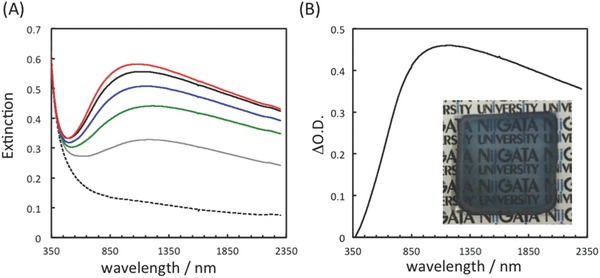
a) Extinction spectra of a TNS/Ag^+^ film following UV‐light irradiation for different times: irradiation time = 0 (dotted), 1 h (gray), 2 h (green), 3 h (blue), 4 h (black), and 5 h (red). b) Differential extinction spectra of a TNS/Ag^+^ film following UV irradiation for 5 h. The inset shows a photograph of the UV‐irradiated TNS/Ag^+^ film after UV‐light irradiation.

A sintered TNS (s‐TNS) film was used as the starting material instead of a TNS/MV^2+^ film. Ag^+^‐containing (TNS/Ag^+^‐2) and NaBH_4_‐treated TNS/Ag^+^‐2 (TNS/AgNP‐2) films were prepared from s‐TNS as described in the Experimental Section. The differential extinction spectra of TNS/AgNP‐2 are shown in Figure S8 (Supporting Information). A clear extinction band is observed up to 1800 nm in the NIR region with λ_max_ ≈ 800 nm. Instead of chemical reduction using NaBH_4_, photocatalytic reduction of Ag^+^ was investigated using the photocatalytic properties of TNS with methanol as the reductant. The changes in extinction spectra and a photograph of TNS/Ag^+^ prepared from TNS/MV^2+^ under 350–375 nm ultraviolet (UV) light (i.e., band gap excitation of TNS) are shown in **Figure** **8**. The characteristic extinction band above ≈450 nm is clearly increased upon UV‐light irradiation, and the color of the films turns to bright blue (Figure 8b, inset), indicating the reduction of Ag^+^ within the interlayers of TNS. Similar spectral changes are also observed using H_2_O as reductant. The differential extinction spectra (Figure 8b) clearly show NIR extinction up to 2000 nm with λ_max_ ≈ 1100 nm. These results indicate that the NIR extinction properties of TNS/AgNP are strongly affected by the experimental procedures. Moreover, these results indicate that further extension of NIR response may be possible through optimization of the preparation procedure. However, the factors that underlie the wavelength extension of the NIR response remain unclear and are currently under investigation in our group.

### 2.6 Photoreaction of TNS/AgNP

**Figure 9:**
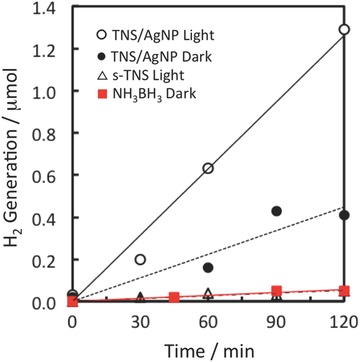
Amounts of hydrogen from aqueous NH_3_BH_3_ (3 mL, 2 × 10^−2^ mol L^−1^) under different conditions: (open circles) TNS/AgNP (*M* = 3) under the 490–900 nm visible‐light irradiation, (solid circles) TNS/AgNP (*M* = 3) under dark conditions, (open triangles) s‐TNS (without AgNPs) under visible‐light irradiation, and (solid squares) NH_3_BH_3_ alone under dark conditions.

Photoassisted H_2_ evolution from NH_3_BH_3_ was investigated[[qv: 3i,j]] as a model reaction because NH_3_BH_3_ shows promise as a next‐generation hydrogen‐storage material.^11^ Time course profiles of H_2_ generation under various conditions are shown in **Figure** **9**. The s‐TNS film, i.e., without AgNP (open triangles), and the aqueous NH_3_BH_3_ solution without any TNS film (solid squares) show almost no H_2_ production under visible‐light irradiation. In contrast, hydrogen is detected under dark conditions for TNS/AgNP (*M* = 3) (solid circle). Visible‐light irradiation of a TNS/AgNP (*M* = 3) film induces further H_2_ production activity (open circles); hydrogen bubbles are immediately generated on the surface of the TNS/AgNP (*M* = 3) upon visible‐light irradiation, as shown in Figure S9 (Supporting Information). Approximately 1.3 µmol of hydrogen was detected in the gas phase following 120 min of visible‐light irradiation (Figure 9) and this amount is approximately three times larger than that for dark conditions (0.41–0.43 µmol). These results clearly indicate that visible‐light irradiated TNS/AgNP (*M* = 3) assists H_2_ generation from NH_3_BH_3_. Visible‐light extinction originating from the LSPR of AgNPs in the TNS layer may enhance the decomposition of NH_3_BH_3_[[qv: 3i,j]] and this material is thus expected to work as a plasmonic catalyst.

Photochemical decomposition of NO into nitrogen oxides (NO*_x_*)^15^ was investigated. TNS/AgNP shows clear photochemical activity for NO decomposition under >290 nm UV‐light irradiation, as shown in Figure S10 (Supporting Information). NO alone and in the presence of s‐TNS shows almost the same level of NO decomposition, indicating that s‐TNS has no photochemical activity for NO decomposition under UV‐light irradiation. In contrast, TNS/AgNP (*M* = 3) shows slight photochemical activity for NO decomposition. Moreover, TNS/AgNP (*M* = 1) shows superior activity for NO decomposition compared to that of NO alone and other TNS films. These results imply that the adsorption of NO on the Ag surface induces its photochemical decomposition.^16^ The difference between TNS/AgNP (*M* = 1) and TNS/AgNP (*M* = 3) may be attributed to the difference in the gas permeability within the interlayer space of the TNSs. Overall, it is thought that the low photochemical activity of both TNS/AgNP (*M* = 1) and TNS/AgNP (*M* = 3) is due to the shape of the films. An increase in the effective surface area is expected to improve the photocatalytic activity of TNS/AgNP, and this is currently under consideration in our group.

## 3 Conclusions

The synthesis of AgNPs within the interlayer spaces of TNSs and their optical and photochemical characteristics were investigated. XRD and EDS analyses indicated that an effective amount of Ag^+^ was intercalated within the TNS layers. The Ag^+^ in the TNS layers was reduced using NaBH_4_, and the color of the film changed to bright blue. Based on the experimental results, we conclude that crystalline AgNPs form within the interlayer space of the TNS films. The AgNP‐containing TNS (TNS/AgNP) film showed intense extinction from the visible‐to‐NIR region and exhibited good stability in air. Repeated AgNO_3_ then NaBH_4_ treatments (*M* = 1–3) increased the characteristic extinction intensity of the material. Much longer wavelength NIR extinction was observed upon the photochemical reduction of Ag^+^ in the TNSs. The TNS/AgNP films exhibit clear photochemical activities for H_2_ production from NH_3_BH_3_ and NO decomposition. These unique properties of TNS/AgNP films demonstrate their promise as a new type of NIR‐responsive material and photocatalyst.

## 4 Experimental Section

*Materials*: The precursor of protonated forms of TNSs (H_0.7_Ti_1.825_O_4_·H_2_O), transparent sintered TNS (s‐TNS) films and methyl‐viologen‐intercalated TNS (TNS/MV^2+^) films were prepared according to well‐established procedures reported previously.^8^, ^9^ Methyl viologen dichloride (MV^2+^ 2Cl^−^; 98%, Aldrich), silver(I) nitrate (AgNO_3_, Wako), sodium tetrahydroborate (NaBH_4_, Junsei Chemical), methanol (Junsei Chemical), and amminetrihydridoboron (NH_3_BH_3_, Aldrich) were used without further purification. Ultrapure water (18.2 MΩ cm^−1^) used in this study was produced by a Milli‐Q water purification system (Direct‐Q 3UV, Millipore). To achieve selective light irradiation, following optical filters were used: Red Rejection 340 (Asahi Spectra), which transmits 300–375 nm light at 50% transmittance (%T); Super Cold 1100 (Asahi Spectra), which transmits 350–1100 nm light at 50%T; Sharp cutoff filter 380 and 490 (Asahi Spectra Co., Ltd.), which enable the removal of <380 and <490 nm light with 50%T, respectively, and a quartz cell filled with water (path length = 5 cm), which enables the removal of NIR to IR light.

*Synthesis of TNS Films* ***—*** *MV^2+^‐Intercalated TNS Films (TNS/MV^2+^)*: An s‐TNS film on a Pyrex glass substrate (≈20 × 20 mm^2^) was immersed in an aqueous solution of MV^2+^ 2Cl^−^ (2 × 10^−4^ mol L^−1^) for 7 h at room temperature, rinsed with ultrapure water, and dried in air at 60 °C in the dark.

*Synthesis of TNS Films* ***—*** *Ag^+^‐Intercalated TNS Films (TNS/Ag^+^)*: A TNS/MV^2+^ film was immersed in an aqueous solution of AgNO_3_ (0.1 mol L^−1^) for 12 h at room temperature, rinsed with ultrapure water, and dried in air at 60 °C in the dark.

*Synthesis of TNS Films* ***—*** *Chemical Reduction of TNS/Ag^+^ and TNS/AgNP Formation*: A TNS/Ag^+^ film was immersed in an aqueous solution of NaBH_4_ (0.1 mol L^−1^) for 0.5 h, then rinsed with water and dried in air at 60 °C under dark conditions. The NaBH_4_ treatments were repeated several times, and the number of repetitions is denoted as *N*.

*Synthesis of TNS Films* ***—*** *Repeated AgNO_3_ and NaBH_4_ Treatments*: A TNS/MV^2+^ film was repeatedly treated with aqueous AgNO_3_ and then NaBH_4_ using the above procedures. The number of repetitions is denoted as *M*.

*Synthesis of TNS Films* ***—*** *Photochemical Reduction of TNS/Ag^+^*: A TNS/Ag^+^ film was irradiated with a 300 W Xe lamp (Asahi Spectra Co., Ltd.) as the light source in 3 mL methanol or water as the reductant. A combination of optical filters (Red Rejection, Super Cold, and cutoff 350) was used for UV‐light irradiation at 350–375 nm (intensity = 5.3 mW cm^−2^).

*Synthesis of TNS Films* ***—*** *Direct Ag^+^‐Intercalated and Chemically Reduced TNS Films (TNS/Ag^+^‐2 and TNS/AgNP‐2)*: An s‐TNS film was immersed in an aqueous solution of AgNO_3_ (0.1 mol L^−1^) for 12 h at room temperature, rinsed with ultrapure water, and dried in air at room temperature in the dark. The obtained Ag^+^‐containing TNS (TNS/Ag^+^‐2) film was then immersed in NaBH_4_, similarly to the procedure used for TNS/Ag^+^.

*Photoreactions* **—** *Hydrogen Evolution From NH_3_BH_3_*: A 6 × 20 mm^2^ sample of TNS/AgNP (*M* = 3) film was placed in a 1 × 1 × ≈5 cm^3^ quartz cell that was then filled with 3 mL of argon‐saturated aqueous NH_3_BH_3_ solution (20 mmol L^−1^). The sample was then irradiated with 490–900 nm visible light (26 mW cm^−2^) using a 300 W Xe lamp equipped with a combination of water, Super Cold, and cutoff 490 filters. The amounts of hydrogen in the gas phase (volume = 1.9 mL) were determined by gas chromatography (GC‐2010 Plus (Shimadzu Co. Ltd.) equipped with a BID‐2010 Plus detector and a SHINCARBON‐ST 80/100 mesh column). Helium was used as the carrier gas.

*Photoreactions* **—** *Photodecomposition of NO*: The photochemical activity for nitrogen monoxide (NO) decomposition was determined using a NO*_x_* analyzer (Yanaco, ECL‐88A). A mixture of 2 ppm NO gas (100 cm^3^ min^−1^) and air (100 cm^3^ min^−1^) was flowed under light irradiation. A TNS/AgNP (*M* = 1 or 3) film (≈20 × 20 mm^2^) was placed on a glass holder plate and set in the center of the reactor. A 450 W high‐pressure mercury lamp equipped with a Pyrex filter was used as the light source for >290 nm light irradiation. Details of the apparatus setup have been previously reported in the literature.[[qv: 10b]]

*Film Characterizations*: XRD analyses were carried out using a desktop X‐ray diffractometer (MiniFlex II, Rigaku) with monochromatized Cu‐K_α_ radiation (λ = 1.5405 Å) operated at 30 kV and 15 mA. EDS was performed using a JED‐2300 apparatus. TEM images were taken using a Topcon EM‐002B operating at an accelerating voltage of 200 kV. XPS was conducted using a JPS‐9000 XPS (JEOL) spectrometer with an Mg‐K_α_ X‐ray line (1254 eV). A multichannel photodetector (MCPD‐3700, Otsuka Electronics) was employed to record extinction spectra at 350–1100 nm. The extinction spectra at visible (380–1100 nm) to wide NIR (850–2000 nm) regions were recorded using a V670 spectrophotometer (JASCO) equipped with a photomultiplier tube and a PdS detector for the visible and NIR regions, respectively. Differential extinction spectra were collected as follows: the spectra were normalized by considering the spectrum of the Ag^+^‐containing film (TNS/Ag^+^) as the background and subtracting it from the spectra obtained after reduction procedures.

## Conflict of Interest

The authors declare no conflict of interest.

## Supporting information

Supplementary
