# Supplementary material for: Preparation of Stable Silver Nanoparticles Having Wide Red‐To‐Near‐Infrared Extinction
Source: Glob Chall. 2018 Feb 21;2(3):1700105. doi: 10.1002/gch2.201700105 (PMC6607257; doi:10.1002/gch2.201700105)
Supplement: Supplementary file 1 — Supplementary [file GCH2-2-1700105-s001.pdf]

Copyright WILEY-VCH Verlag GmbH & Co. KGaA, 69469 Weinheim, Germany, 2018.

Global Challenges
Open Access

Supporting Information

for *Global Challenges,* DOI: 10.1002/gch2.201700105

Preparation of Stable Silver Nanoparticles Having Wide Red-To-Near-Infrared Extinction

*Shiori Kawamura, Kazuki Matsubara, Sotaro Sakai, Kazuhisa Sasaki, Masataro Saito, Kenji Saito, Masayuki Yagi, Wataru Norimatsu, Ryo Sasai, Michiko Kusunoki, Miharu Eguchi, Shu Yin, Yusuke Asakura, and Tatsuto Yui**

Preparation of Stable Silver Nanoparticles having Wide Red-to-Near Infrared Extinction

Shiori Kawamura,[a)] Kazuki Matsubara,[a)] Sotaro Sakai,[a)] Kazuhisa Sasaki,[a)] Masataro Saito,[a)] Kenji Saito,[a)] Masayuki Yagi,[a)] Wataru Norimatsu,[b)] Ryo Sasai,[c)] Michiko Kusunoki,[d)] Miharu Eguchi,[e)] Shu Yin[f)], Yusuke Asakura[f)], and Tatstuto Yui [a),]*

a. Department of Materials Science and Technology, Faculty of Engineering, Niigata University, 8050 Ikarashi‒2, Niigata 950‒2181, Japan. E‒mail: ttt_yui@mac.com

b. Department of Materials Chemistry, Graduate School of Engineering, Nagoya University, Furo–cho, Chikusa–ku, Nagoya–shi, Aichi–ken, 464–8603, Japan

c. Department of Physics and Materials Science, Interdisciplinary Graduate School of Science and Engineering, Shimane University, 1060, Nishi–kawatsu–cho, 690–8504 Matsue, Japan

d. Institute of Materials and Systems for Sustainability, Nagoya University, Furo–cho, Chikusa–ku, Nagoya–shi, Aichi–ken, 464–8603, Japan

e. Electronic Functional Materials Group, Polymer Materials Unit, National Institute for Materials Science (NIMS), 1-1 Namiki, Tsukuba, Ibaraki 305-0044, Japan

f. Institute of Multidisciplinary Research for Advanced Materials, Tohoku University, 2-1-1, Katahira, Aoba-ku, Sendai 980-8577, Japan

*E-mail: ttt_yui@mac.com

Table S1: Diffraction angles (2θ), *d* values, and full width at half maximum (FWHM) values of the *d*(002) signals in the XRD profiles, and the estimated clearance space (CLS) values of various TNS films.

	2θ / °	*d*(002) / nm	FWHM / °	CLS[a)] / nm
TNS/MV^{2+}	7.8	1.13	0.11	0.38
TNS/Ag^{+}	8.7	1.01	0.20	0.26
TNS/AgNP (N = 1)	8.5	1.04	0.25	0.29

a) Clearance space can be defined as the value obtained by subtraction of the thickness of one TNS sheet (0.75 nm)[1-4] from the observed *d*(002) distance.

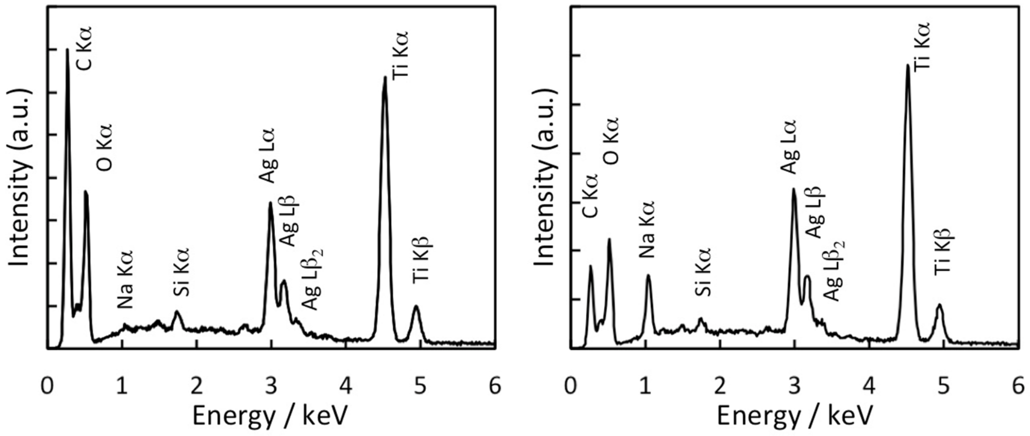

Figure S1. EDS spectra of $AgNO_3$-treated (TNS/Ag^+) (a) and $NaBH_4$-treated TNS/Ag^+ (TNS/AgNP, N = 1) (b) films.

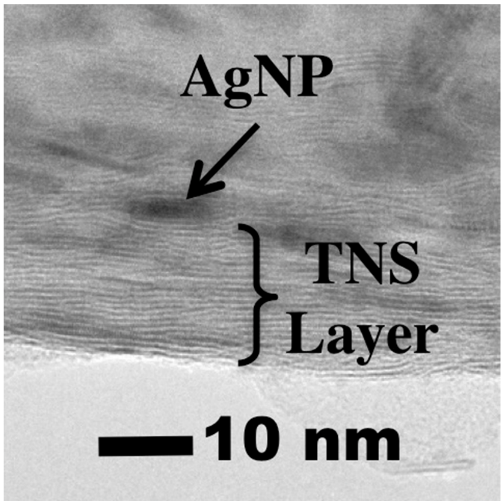

Figure S2. TEM image of $NaBH_4$-treated TNS/Ag^+ (TNS/AgNP, N = 1).

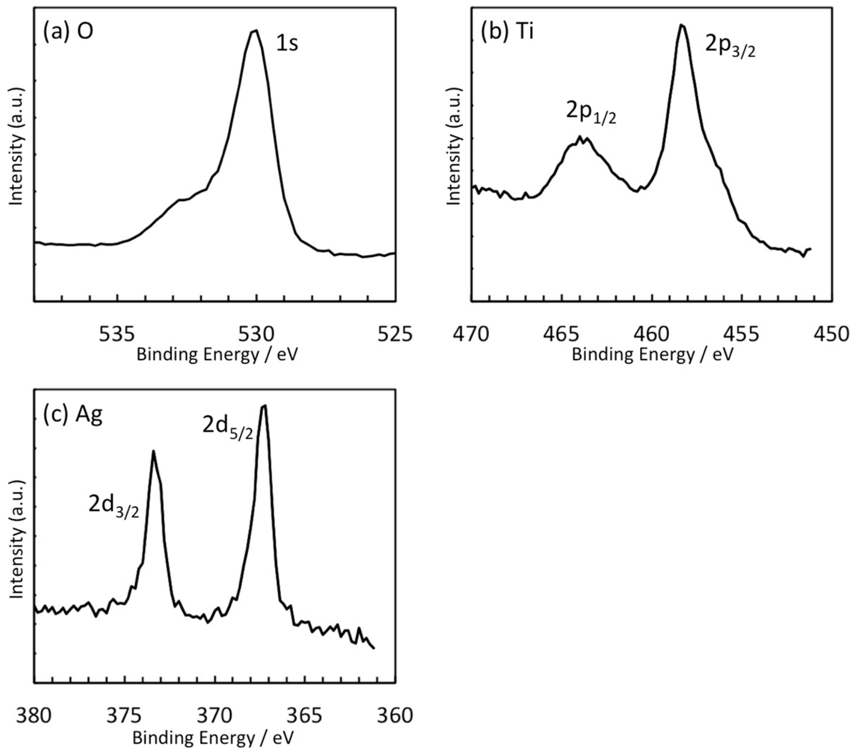

Figure S3. XPS spectra of the $NaBH_4$-treated TNS/Ag^+ (TNS/AgNP, N = 1) film: (a) O 1s, (b) Ti 2p, and (c) Ag 2d.

Table S2: XPS spectra data of the $NaBH_4$-treated TNS/Ag^+ (TNS/AgNP, N = 1) film.

	**O**	**Ti**		**Ag**	
Orbital	1s	$2p_{3/2}$	$2p_{1/2}$	$3d_{5/2}$	$3d_{3/2}$
Energy / eV	530.1	458.3	464.0	367.3	373.3
Previous Report[5] / eV	529.6 ± 0.2	458.1 ± 0.2	463.8 ± 0.2	366.9 ± 0.2	373.0 ± 0.2

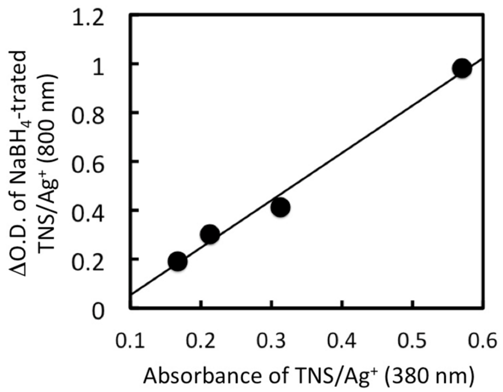

Figure S4. Relationship between the absorption intensities at 380 nm for TNS/Ag^{+} (absorption by TNS) and the extinction intensities at 800 nm for $NaBH_4$-trated TNS/Ag^{+} (TNS/AgNP, N = 1) with different amounts of TNS on glass substrates.

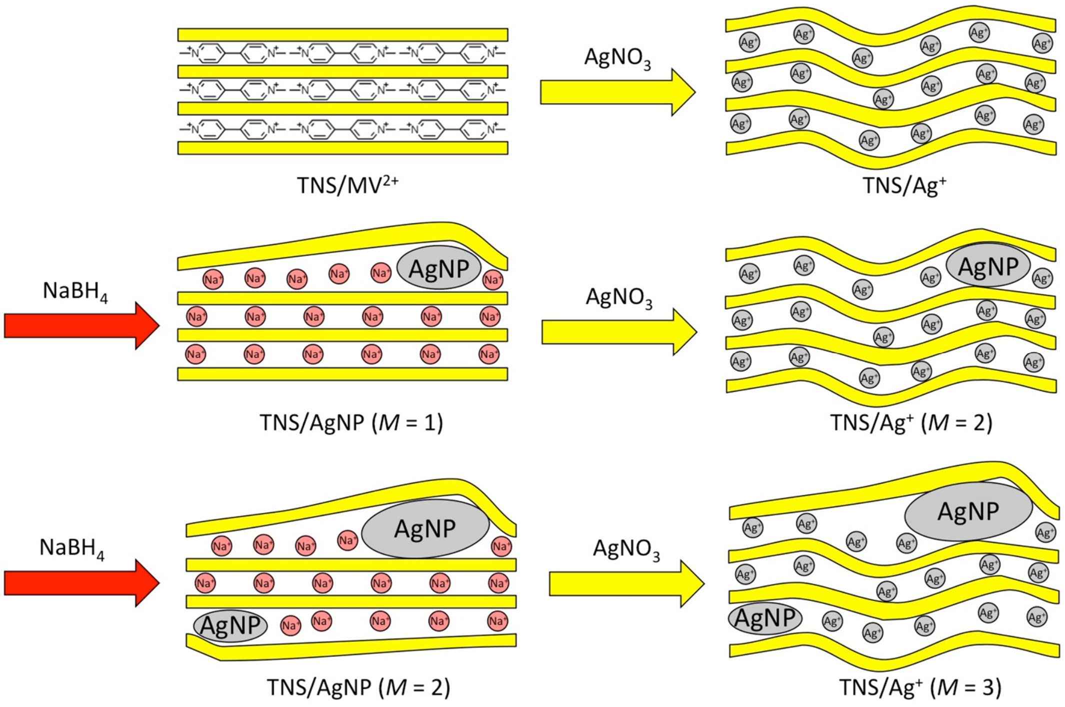

Figure S5. Proposed schematics of TNSs containing different cations.

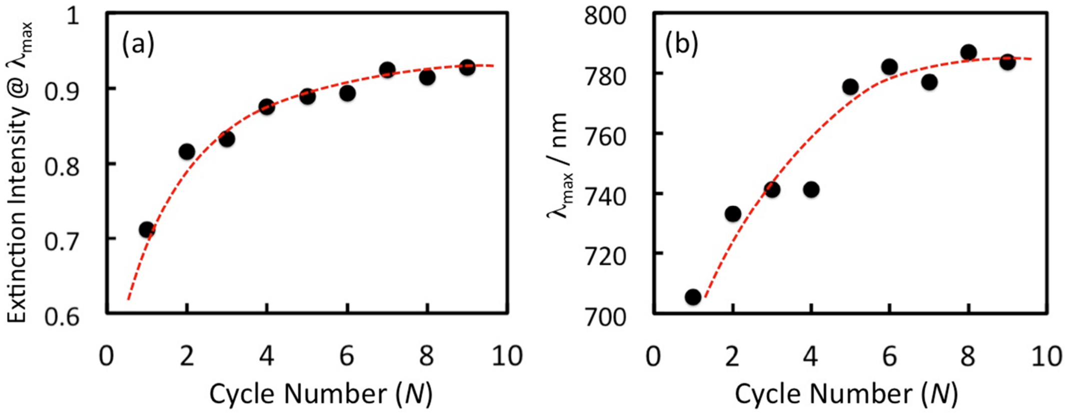

Figure S6. Relationship between the number of repeated $NaBH_4$ treatments (N) and extinction intensity (a) and maximum wavelength (b) of TNS/AgNP.

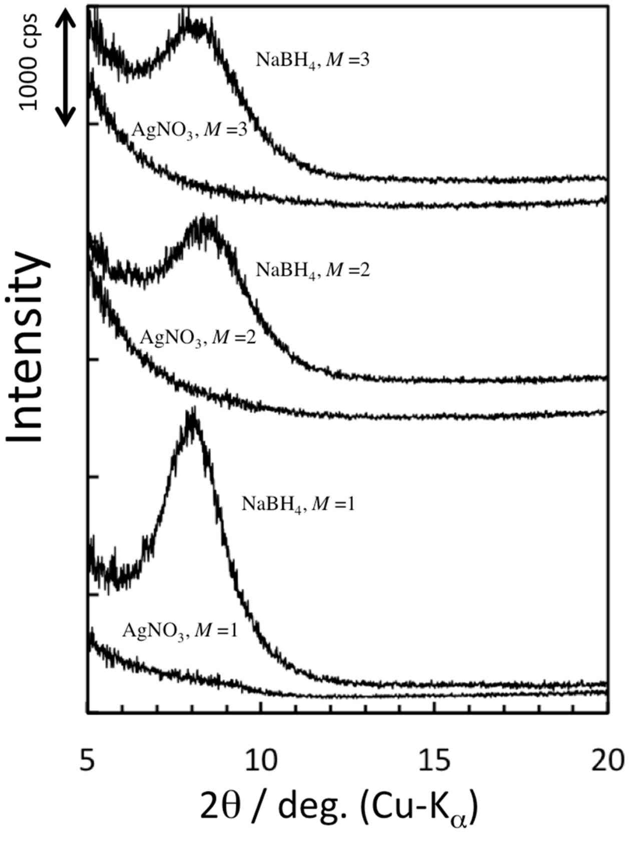

Figure S7. XRD profiles following repeated $AgNO_3$ then $NaBH_4$ treatment (M = 1–3).

Table S3: Amounts of Silver and Sodium Atoms (% CEC) in TNS Films through Repeatable $AgNO_3$ and $NaBH_4$ Treatments

Cycle Number (*M*)	Treatment	Ag	Na
1	$AgNO_3$	71.9 ± 3.1	5.3 ± 1.2
	$NaBH_4$	73.1 ± 5.4	58.1 ± 4.4
2	$AgNO_3$	158.6 ± 4.3	4.6 ± 2.0
	$NaBH_4$	152.3 ± 5.8	46.5 ± 6.0
3	$AgNO_3$	252.1 ± 6.8	4.1 ± 1.9
	$NaBH_4$	233.8 ± 4.4	47.7 ± 4.6

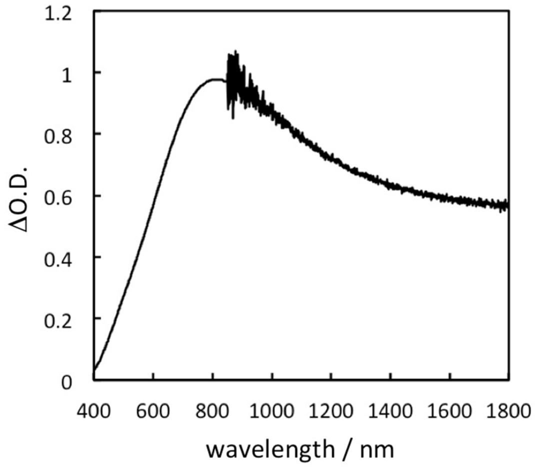

Figure S8. Differential extinction spectra of $NaBH_4$-treated TNS/Ag^+-2 (TNS/AgNP-2).

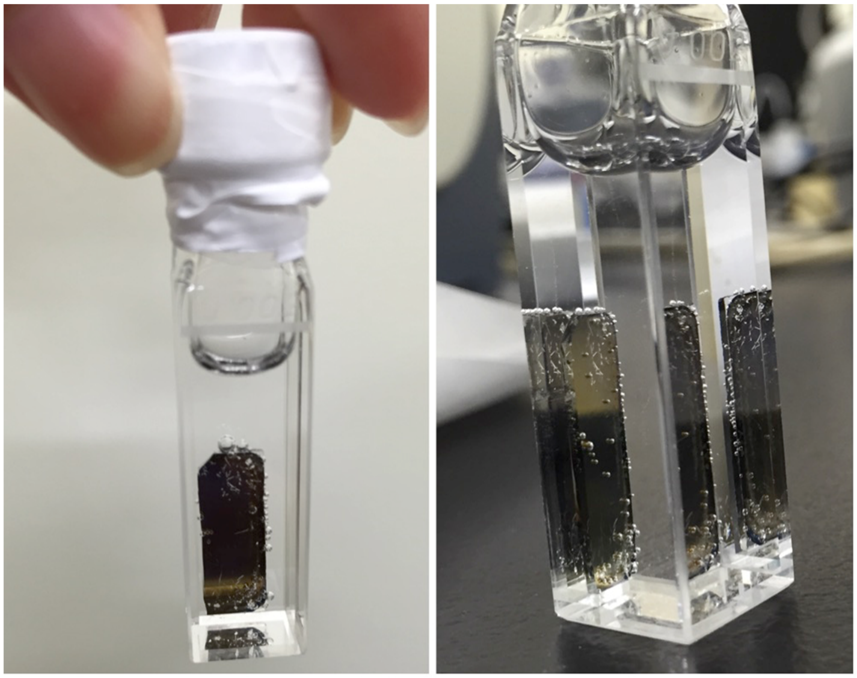

Figure S9. Photographs of TNS/AgNP (*M* = 3) in aqueous NH_3BH_3 upon visible-light irradiation.

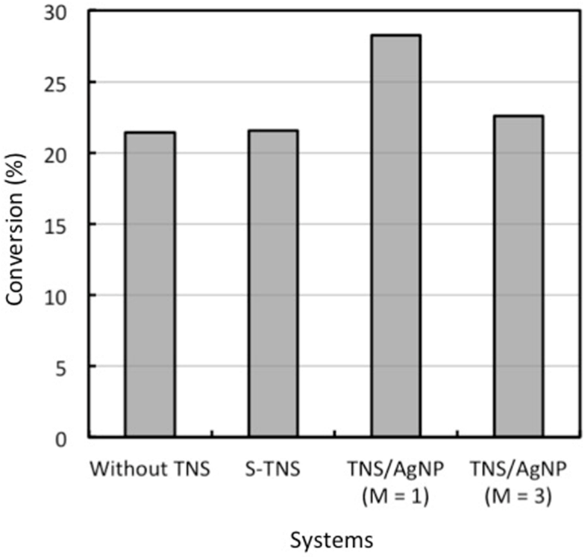

Figure S10. Photochemical NO conversions using various systems.

References

1. Sasaki, T.; Watanabe, M.; Hashizume, H.; Yamada, H.; Nakazawa, H. Macromolecule-like Aspects for a Colloidal Suspension of an Exfoliated Titanate. Pairwise Association of Nanosheets and Dynamic Reassembling Process Initiated from It. *J. Am. Chem. Soc.* 1996, *118*, 8329-8335.

2. Sasaki, T.; Watanabe, M. Osmotic Swelling to Exfoliation. Exceptionally High Degrees of Hydration of a Layered Titanate. *J. Am. Chem. Soc.* 1998, *120*, 4682-4689.

3. Tanaka, T.; Ebina, Y.; Takada, K.; Kurashima, K.; Sasaki, T. Oversized Titania Nanosheet Crystallites Derived from Flux-Grown Layered Titanate Single Crystals. *Chem. Mater.* 2003, *15*, 3564-3568.

4. Wang, L.; Sasaki, T. Titanium Oxide Nanosheets: Graphene Analogues with Versatile Functionalities. *Chem. Rev.* 2014, *114*, 9455-9486.

5. Zhou, Y.; Ma, R.; Ebina, Y.; Takada, K.; Sasaki, T. Multilayer Hybrid Films of Titania Semiconductor Nanosheet and Silver Metal Fabricated via Layer-by-Layer Self-Assembly and Subsequent UV Irradiation. *Chem. Mater.* 2006, *18*, 1235-1239.
